# Effect of Membrane Properties on the Carbonation of Anion Exchange Membrane Fuel Cells

**DOI:** 10.3390/membranes11020102

**Published:** 2021-01-31

**Authors:** Yiwei Zheng, Lyzmarie Nicole Irizarry Colón, Noor Ul Hassan, Eric R. Williams, Morgan Stefik, Jacob M. LaManna, Daniel S. Hussey, William E. Mustain

**Affiliations:** 1 Department of Chemical Engineering, Swearingen Engineering Center, University of South Carolina, Columbia, SC 29208, USA; yiweiz@email.sc.edu (Y.Z.); lyzmarie@email.sc.edu (L.N.I.C.); ulhassan@email.sc.edu (N.U.H.); 2 Department of Chemistry and Biochemistry, University of South Carolina, Columbia, SC 29208, USA; ericrw@email.sc.edu (E.R.W.); stefik@mailbox.sc.edu (M.S.); 3 National Institute for Standards and Technology, Gaithersburg, MD 20899, USA; jacob.lamanna@nist.gov (J.M.L.); daniel.hussey@nist.gov (D.S.H.)

**Keywords:** anion exchange membrane, fuel cell, CO_2_, carbonation, conductivity

## Abstract

Anion exchange membrane fuel cells (AEMFC) are potentially very low-cost replacements for proton exchange membrane fuel cells. However, AEMFCs suffer from one very serious drawback: significant performance loss when CO_2_ is present in the reacting oxidant gas (e.g., air) due to carbonation. Although the chemical mechanisms for how carbonation leads to voltage loss in operating AEMFCs are known, the way those mechanisms are affected by the properties of the anion exchange membrane (AEM) has not been elucidated. Therefore, this work studies AEMFC carbonation using numerous high-functioning AEMs from the literature and it was found that the ionic conductivity of the AEM plays the most critical role in the CO_2_-related voltage loss from carbonation, with the degree of AEM crystallinity playing a minor role. In short, higher conductivity—resulting either from a reduction in the membrane thickness or a change in the polymer chemistry—results in faster CO_2_ migration and emission from the anode side. Although this does lead to a lower overall degree of carbonation in the polymer, it also increases CO_2_-related voltage loss. Additionally, an *operando* neutron imaging cell is used to show that as AEMFCs become increasingly carbonated their water content is reduced, which further drives down cell performance.

## 1. Introduction

Recently, anion exchange membrane fuel cell (AEMFC) performance and stability have been enhanced to the point where their future deployment in real applications can be seriously considered. AEMFC peak power densities have been reported as high as 3.4 W/cm^2^ operating on H_2_/O_2_ gas feeds [1] and 1.7 W/cm^2^ operating on H_2_/CO_2_-free air [2]. In addition, multiple groups have reported 500+ h stability at low degradation rates (5–10%) [3,4,5] and one recent study showed durability over 2000 h with only a 3.65% voltage decay [2]. However, there remains one very serious issue that must be addressed: performance loss caused by exposure of operating cells to carbon dioxide, typically from the ambient air cathode feed.

When CO_2_ enters an operating AEMFC, it reacts with the OH^−^ anions in the polymer as well as those being continuously produced in the cathode from oxygen reduction (Equation (1)) to form (bi)carbonates [6,7,8] (Equations (2) and (3)).

(1) $$O_{2}+2H_{2}O+4e^{-}\to 4OH^{-}$$

(2) $$OH^{-}+CO_{2}↔HCO_{3}^{-}$$

(3) $$HCO_{3}^{-}+OH^{-}↔CO_{3}^{2-}+H_{2}O$$

Once the (bi)carbonate anions are formed, there are three primary mechanisms [9,10,11,12], all tied to the movement of the (bi)carbonate anions to the anode, that lead to a reduction in the operating voltage of the cell, which can amount to as much as 400 mV [13]. First, because hydrogen does not directly react with the (bi)carbonate anions arriving at the anode at typical operating potentials, they accumulate in the anode over time, lowering the pH. The drop in the anode pH leads to a thermodynamically driven increase in the anode potential (Δ*V_Nernst_*, typically 160–210 mV), decreasing the operating voltage. Second, accumulated (bi)carbonates lead to increased anode charge transfer resistance (Δ*R_ctHOR_*) and higher kinetic overpotentials (Δ*V_ctHOR_* = *i·R_ctHOR_*, typically 50–100 mV). Third, (bi)carbonates have lower mobility than hydroxide, which lowers ionic conductivity and increases the area-specific resistance (Δ*ASR*), leading to a corresponding voltage loss (Δ*V_ASR_* = *i*⋅Δ*ASR*, typically 10–20 mV). It is important to note that the impact of the increase in the ASR is generally a minor contributor (<10%) to the total CO_2_-related operating voltage loss, which is dominated by the kinetic and thermodynamic effects discussed above [9,11]. Taking the three mechanisms for CO_2_-related voltage loss into consideration, the operating voltage for an AEMFC upon exposure to CO_2_ can be described by Equation (4) [9]:

(4) $$V_{cell}=V_{OCV}-i\left(R_{\Omega ,OH}+R_{ctORR}+R_{mtORR}+R_{ctHOR}\right)-{\Omega V}_{Nernst}\;-i\left(\Omega ASR+{\Omega R}_{ctHOR}\right)$$

where *V_OCV_* is the open-circuit voltage, *i* is the cell current, *R_Ω,OH_* is the Ohmic resistance to ion transport, *R_ctORR_* and *R_ctHOR_* are the charge transfer resistances for oxygen reduction reaction and hydrogen oxidation reaction, respectively, and *R_mtORR_* is the mass transport resistance for oxygen reduction reaction.

Recently, there have been several experimental [9,14,15,16,17,18] and modeling [11,19,20,21] studies focused on quantifying the effect of CO_2_ on operating AEMFCs. Early work in this area suggested that the cumulative CO_2_-related voltage losses might be too large to overcome and it might be necessary to almost completely remove carbon dioxide from the cathode air feed [9,15,16], which could be quite expensive and add system complexity. However, recent studies have identified several pathways by which the CO_2_-related voltage penalty can mitigated [9,10,13,19] such as increasing the AEMFC operating current density, cell temperature and hydration level as well as decreasing the cathode flowrate. In high-performing AEMFCs, operating conditions have been found that minimize the CO_2_-related voltage loss to only 30 mV with 5 ppm CO_2_ in the cathode feed and 182 mV with 400 ppm CO_2_ in the cathode feed [10]. However, there are no studies in the present literature that have systematically studied the effect of the AEM itself and there does not exist a fundamental link between the AEM properties and the degree of AEMFC carbonation.

That being said, a tremendous amount of literature has been generated regarding membrane preparation and the investigation of the backbone chemistry [22,23,24], headgroup chemistry [25,26,27], and structure [28,29,30] on their electrochemical and mechanical properties [31,32,33]. Those electrochemical properties (especially hydroxide conductivity) are directly related to cell Ohmic resistance as well as the water uptake (WU) and transport. These can have an impact on the uptake of CO_2_ and transport of carbonate in AEMFCs. Additionally, membrane thickness can be used to manipulate the transport of both ions and water in AEMFCs [34]. Because it has been shown that the accumulation and release of CO_2_ is related to cell hydration and anode pH, it is very likely that the wise selection of AEMs may further allow AEMFCs to resist carbonation. Therefore, it is important to explore the impact of AEM physical and electrochemical properties on the tolerance of operating AEMFCs to the presence of CO_2_ in the cathode feed. 

In this study, we investigate the carbonation of AEMFCs assembled from a series of very high-performing AEMs from the literature with different chemistries and properties, including high-density polyethylene with a benzyltrimethylammonium cation (HDPE-BTMA) [35], low-density polyethylene with a benzyltrimethylammonium cation (LDPE-BTMA) [35], poly (norbornene) copolymers of GT72-5 [36], GT78-15 [1], GT64-15 [37], poly(aryl piperidinium) copolymer that possesses a terphenyl chain (PAP-TP-85) [38] (the full names for each AEM are defined in the Experimental section). After cell startup and break-in, each of the AEMs are exposed to 400 ppm CO_2_. During this time, the dynamics of cell carbonation are observed and the degree of carbonation and magnitude of the CO_2_-related voltage loss are quantified. The results allow the effect of several variables on AEM/AEMFC carbonation to be elucidated, including: AEM thickness, conductivity and crystallinity. Lastly, the carbonation of a 15 µm HDPE-BTMA AEM—with highest CO_2_-related voltage loss—was investigated *operando* by neutron imaging in order to quantify the effect of adding CO_2_ to the amount of liquid water in the membrane electrode assembly (MEA), which is the first time that the water content of a carbonated AEMFC has been imaged.

## 2. Experimental

### 2.1. Electrode Preparation

The electrodes in this work were prepared using a method that has been detailed in our previous publications [10,39,40,41]. Briefly, the anode catalyst was PtRu (weight fraction of 60%) supported on Vulcan XC-72R (Alfa Aesar HiSPEC 10000, 2:1 ratio of Pt:Ru by mass, Alfa Aesar, Tewksbury, MA, USA). The cathode catalyst was Pt (weight fraction of 40%) supported on Vulcan XC-72R (Alfa Aesar HiSPEC 4000). The ionomer was a benzyltrimethylammonium-functionalized ethylene tetrafluoroethylene (ETFE-BTMA) solid powder anion exchange ionomer (AEI, ion-exchange capacity IEC = 1.24 mmol g^-1^) [42].

Electrode preparation was initiated by placing the ionomer into a mortar and grinding it with a pestle by hand for 10 min. The catalyst powder, Vulcan carbon (XC-72R, Cabot, Alpharetta, GA, USA), and Millipore deionized water (Millipore Sigma Milli-Q^®^, Burlington, MA, USA, DI, 18.2 MΩ cm resistivity) were added to the mortar and ground for an additional 10 min. The mass fraction of AEI in the catalyst layer was always 0.20 and the mass fraction of carbon was maintained at 0.48 for both electrodes. Next, the catalyst-AEI slurry was transferred to a centrifuge tube. Isopropyl alcohol (IPA, Fisher Scientific, Waltham, MA, USA) solvent was added until the IPA:DI water ratio was 9:1 by volume. The mixture was sonicated (Fisher Scientific FS30H, Waltham, MA, USA) for 60 min. The water in the ultrasonic bath was maintained below 5 °C to avoid any AEI degradation and to avoid catalyst agglomeration. The ink dispersions were sprayed onto Toray TGP-H-060 (Fuel Cell Store, College Station, TX. USA) gas diffusion layers with PTFE wetproofing (weight fraction of 5%) with an Iwata Eclipse HP-CS (ANEST IWATA, Cincinnati, OH, USA) to create gas diffusion electrodes (GDEs). For spraying, the carrier gas was 103 kPa (15 psig) ultra high purity (UHP) N_2_. 

### 2.2. Anion Exchange Membrane Fuel Cell (AEMFC) Assembly and Break-In Procedure

**Figure 1:**
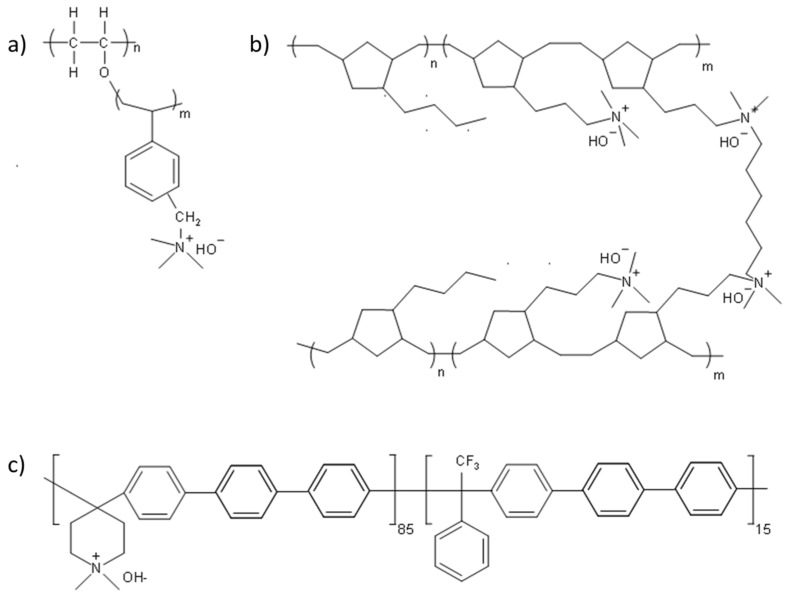
The structure of anion exchange membranes (AEMs) used in this study (**a**) high-density polyethylene polymer with a benzyltrimethylammonium cation (HDPE-BTMA) and low-density polyethylene polymer with a benzyltrimethylammonium cation (LDPE-BTMA), (**b**) poly (norbornene) copolymer of GT64-15, GT72-5 and GT78-15 and (**c**) poly(aryl piperidinium)-based polymer with a terphenyl chain (PAP-TP-85) [1,3,4,29,39,40,41,42].

Eight AEMs were investigated in this study. Their structures and properties are summarized in Figure 1 and Table 1, respectively. The first two AEMs were radiation-grafted 15 μm or 25 μm low-density polyethylene with a benzyltrimethylammonium (LDPE-BTMA) stationary cation [43]. The third AEM was a 15 μm radiation-grafted high-density polyethylene polymer with a BTMA cation (HDPE-BTMA) [35]. The fourth AEM was a 15 μm poly(aryl piperidinium) (PAP)-based copolymer. Both parts of the copolymer have primary repeat group that possesses a terphenyl (TP) chain. The AEM used here is denoted as PAP-TP-85, which means that a mole fraction of 0.85 of the copolymer contains the piperidinium group and a mole fraction of 0.15 does not [38]. 

The final family of four AEMs were based on cross-linked tetra-block poly(norbornene) (PNB) copolymers [36,37]. These AEMs were made from a combination of halogenated and non-halogenated monomers and a *N,N,N’*, *N*’-tetramethyl-1, 6-hexanediamine (TMHDA) crosslinker. In the following discussion, these AEMs are denoted as GTXX-YY where XX indicates the percentage of the halogenated monomer and YY provides the mass fraction (in %) of the TMHDA. Therefore, a GT78-15 AEM comprises 78% halogenated monomer and 15% crosslinker. Two GT78-15 (10 μm and 20 μm thickness), one GT64-15 (10 μm) and one GT72-5 (10 μm) AEMs were studied.

Before cell assembly, the GDEs and AEMs were soaked in separate 1 M aqueous KOH solutions (made from Fisher Chemical-certified ACS pellets and DI water) for 60 min, exchanging the solution twice during this time. After the 1 h soak, excess KOH was removed from the GDEs and AEMs and they were pressed together in the cell with no prior hot pressing to form membrane electrode assemblies (MEAs). The MEAs were loaded into 5 cm^2^ Scribner hardware between two single pass serpentine flow graphite plates. Each MEA was sealed and compressed with 6 mil (152 μm) polytetrafluoroethylene (PTFE) at 5.1 N∙m torque, resulting in 20–25% pinch. An 850e Scribner Fuel Cell Test Station was used to control the gas stream dew points, cell temperature, gas flowrates and the operating current density.

Before CO_2_ measurements were made, all cells underwent a break-in procedure. First, a cell was brought to its operating temperature under N_2_ flow on both sides of the cell at 100% relative humidity (RH). Then, the feed gases were switched to UHP H_2_ and O_2_ (Airgas) at the anode and cathode, respectively. Next, the cell was operated potentiostatically, stepwise from 0.7 to 0.3 V (0.1 V steps, held for a minimum of 30 min at each step) as the reacting gas dew points were optimized [39,40]. Dew point optimization was performed by iteratively changing the anode and cathode dew point every 5–10 min, allowing the cell to equilibrate, and analyzing the cell’s performance through forward and reverse linear sweeps, voltage holds, current holds, current interrupt, and high-frequency resistance (HFR) measurements. The optimized dew points were selected to be the set that maximized the operating voltage at constant current density without sacrificing membrane hydration (determined by the HFR measurement). The optimized reacting gas dew points were very repeatable from cell to cell for the same membrane; but to keep consistent hydration level across all cells, the dew points were typically maintained as: (i) 72 °C at the anode and 72 °C at the cathode for an AEMFC operating at 80 °C or (ii) 50 °C at the anode and 52 °C at the cathode for an AEMFC operating at 60 °C. Following the optimization of the reacting gas dew points, the cells were operated galvanostatically at 0.2 A cm^−2^. They were allowed to equilibrate for at least 30 min before CO_2_ exposure was initiated. Multiple cells were constructed and tested for each measurement.

### 2.3. AEMFC Carbon Dioxide Measurements

Following the break-in procedure and 30 min equilibration, the cell current was maintained and 400 ppm CO_2_ was added to the UHP O_2_ cathode stream. CO_2_ was added to O_2_ in lieu of air in order to simplify observations because air has additional O_2_ mass transport implications (e.g., N_2_ dilution) during cell operation, which is largely eliminated by utilizing O_2_ as the reacting gas. The flowrates for O_2_ and H_2_ were 1.0 L min^−^^1^. Typically, after CO_2_ addition, the cell was operated for 10 min, which was much longer than the time required to reach quasi-steady-state operation (typically < 5 min). After 30 min operation at constant current, CO_2_ was removed from the gas stream and the cell was allowed to decarbonate for an initial 10 min. After this, the cell was fully decarbonated by lowering the cell voltage to 0.1 V for 2 min, after which no CO_2_ emission was measured in the anode stream and the operating voltage returned to the value observed before CO_2_ was added. When CO_2_ was fed to the cathode, the concentration of CO_2_ emitted from the anode and cathode was constantly monitored in real time using a PP Systems WMA-5 non-dispersive infrared CO_2_ gas analyzer (a water trap was placed in-line before the WMA-5 in order to preserve the unit and its calibration). 

### 2.4. Wide Angle X-ray Scattering Analysis

X-ray experiments were conducted using a SAXSLab Ganesha (SAXSLab, Holyoke, MA, USA) at the South Carolina SAXS Collaborative. A GeniX 3D (Xenocs, Grenoble, France) microfocus source was used with a copper target to produce a monochromatic beam with a wavelength of 0.154 nm. The instrument was calibrated just prior to use with the National Institute of Standards and Technology reference material, 640 d silicon powder with the peak position of 2𝜃 = 28.44°, where 2𝜃 refers to the total scattering angle. A Pilatus 300 k detector (Dectris, Baden-Daettwil, Switzerland) was used to collect the two-dimensional (2D) scattering pattern with nominal pixel dimensions of 172 µm × 172 µm. The SAXS data were acquired with an X-ray flux of~40 million photons per second incident upon the sample at a sample-to-detector distance of 104.5 mm. The 2D images were azimuthally integrated to yield the scattering vector and intensity. Crystalline and amorphous signal were deconvolved by fitting the former to a linear baseline for integration. Integrals were performed using custom software coded in MATLAB (Version 2020b, Mathworks, Natick, MA, USA). Percent crystallinity was calculated based upon the following relationship: $\frac{X_{c}}{X_{c}+X_{a}}$ where *X_c_* and *X_a_* refer to the total integral intensity of the crystalline and amorphous regions, respectively. Additional details and plots of the raw data are provided in the Supporting Information. 

### 2.5. Neutron Imaging Cell and Operation

The *operando* neutron scattering imaging experiments were conducted at the NIST (National Institute for Standards and Technology) Center for Neutron Research (NCNR). A diagram showing the dimensions of the flowfields for these *operando* measurements is shown in Figure S1 in the Supporting Information. The GDEs used in these experiments were identical to the ones described above, only cut to a smaller size after spraying. Before cell assembly, the membranes and GDEs were treated identically to above. The main difference was the cell hardware, which consisted of a gold-plated combination current collector and flow fields with a parallel flow pattern and active area of 2.5 cm^2^. Again, cells were assembled with 6-mil gaskets to achieve 20-25% pinch. The cells were then humidified and broken in under the same protocol used for the 5 cm^2^ cells. After stably running a cell for more than 8 h, 1% CO_2_ was introduced into cathode gas stream and the cell was run stably for another 8 h. The neutron images were collected on the BT-2 beamline at the NCNR [44,45], and captured with an intensified macroscope detector capable of determining the centroid of emitted scintillation light [46]. For collimation, the L/D was 6000 along the through plane direction. Since the center of the test section was about 3 cm from the detector, the full-width half maximum of the geometric blur was ~2.5 μm. To align the *operando* fuel cell along the beam to yield 1 μm resolution would require an angular resolution on the rotation state of better than 0.005 degrees, which was within our state angular resolution of 0.001 degrees. For each fuel cell data point, about 4 h of centroid data were acquired, corresponding to a live time of about 20 min.

## 3. Results and Discussion

### 3.1. Influence of Anion Exchange Membrane (AEM) Thickness on AEMFC Performance with 400 ppm Cathode CO_2_

In the absence of CO_2_, AEMFC performance generally increases as the membrane thickness decreases due to a combination of decreased ohmic resistance and increased water diffusivity [34,47,48,49]. It is also known that the water balance between the anode and cathode is extremely important for AEMFC performance as either cathode dryout or anode flooding can severely compromise FC stable operation [39]. In our previous publications [10], we also showed that FC water content has an effect on the CO_2_ uptake into the cell and that a considerable fraction of the carbonation dynamics rely on what is happening in the anode while the AEM can be a reservoir for carbonate. Therefore, despite the fact that increasing membrane thickness should increase the cell Ohmic resistance, it does not naturally follow that increasing the AEM thickness will increase the CO_2_-related voltage losses. 

**Figure 2:**
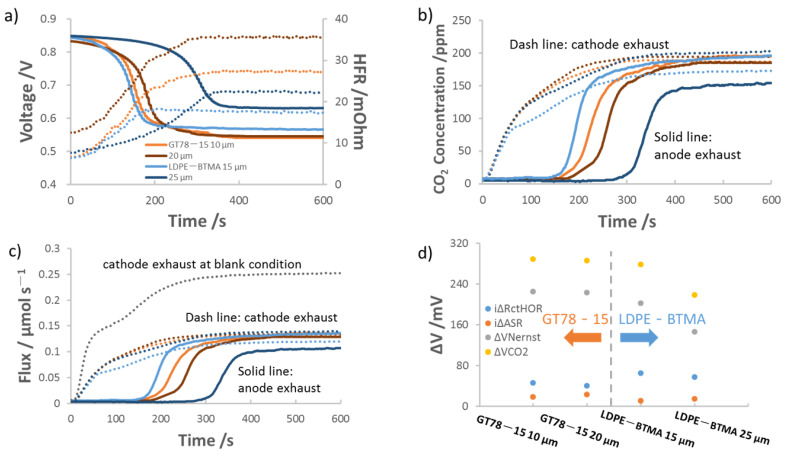
Effect of AEM thickness on anion exchange membrane fuel cell (AEMFC) carbonation and performance loss. The AEMFCs were operated at 0.2 A/cm^2^ and 76 °C/76 °C/80 °C with 10 μm, 20 μm GT78-15 and 15 μm, 25 μm LDPE-BTMA AEMs, 400 ppm CO_2_ fed to cathode at t = 0 s, and 1 L/min H_2_/O_2_ flowrates. The anode catalyst loading was 0.5 mg_Pt_ cm^−2^ ± 0.1 mg_Pt_ cm^−2^. The cathode catalyst loading was 0.6 mg_Pt_ cm^−2^ ± 0.1 mg_Pt_ cm^−^^2^. (**a**) Voltage loss (solid lines) and high-frequency resistance (HFR) (dotted lines) following the introduction of CO_2_ into the cathode; (**b**) concentration of CO_2_ in the anode (solid lines) and cathode (dotted lines) effluent streams; (**c**) CO_2_ flux leaving the anode and cathode; (**d**) deconvolution of the CO_2_-related voltage losses.

The effect of AEM thickness on the AEMFC carbonation is shown in Figure 2. Figure 2 presents representative data from 2 sets of AEMs: 10 μm/20 μm GT78-15 and 15 μm/25 μm LDPE-BTMA. Figure 2a is the dynamic measurement of the voltage decrease and HFR increase for the AEMFCs deploying the AEMs above as CO_2_ was introduced into the system. At t = 0 s, CO_2_ was introduced to the cathode, and in all cases the voltage decreased and HFR increased. After ca. 5 min, a steady state voltage and HFR were reached and the rate in which the cell is taking up CO_2_ at the cathode was equal to the rate of CO_2_ emission at the anode. For both membrane types, a thinner membrane led to increased total voltage loss and a lower Δ*ASR*.

Figure 2b shows the dynamic measurements for the concentration of CO_2_ in the exhaust of the anode and cathode, which is transformed into molar flux in Figure 2c. In all cases, there was a time lag from the time that CO_2_ was fed to the cathode and the time that CO_2_ was observed purging out of the anode. This time lag is the result of carbonate accumulation in the anode, as discussed in the introduction, which lowers the anode pH until the reverse of Equations (2) and (3) are favorable and CO_2_ is emitted. Within the same AEM chemistry, thinner membranes had lower lag times. This is most likely because thinner AEMs have fewer charge-carrying groups, allowing carbonation and equilibration to occur faster. The increase in charge carrying groups means that thicker AEMs can uptake more CO_2_, which is confirmed by calculations of the total amount of carbonate (*N_CO2_*) in Table 2. Details for the calculation of *N_CO2_* from the data in Figure 2c as well as degree of carbonation (DOC) are provided in the Supporting Information.

From the carbonation data in Figure 2 and the CO_2_-release data in Figures S3–S6, it is possible to deconvolute the overall voltage loss from CO_2_ into the contributions from the three main mechanisms discussed in the introduction: Δ*ASR*, Δ*V_Nernst_* and Δ*R_ctHOR_*. The process to perform the deconvolution is described in the Supporting Information and detailed in our previous publication [9] and the results are shown in Figure 2d and Table 2. As expected from the carbonation data in Table 2, where thicker membranes contained more carbonates, thicker membranes had both higher Δ*ASR* and higher overall resistance. However, AEMFCs with thicker membranes experienced less Δ*R_ctHOR_* and Δ*V_Nernst_*, suggesting that AEMFCs with thicker AEMs may have less carbonate accumulated at the anode side or at least a longer distance over which the carbonate concentration gradient is relaxed.

Though the overall trends using both AEMs were the same, the degree of carbonation and the extent to which Δ*ASR*, Δ*R_ctHOR_* and Δ*V_Nernst_* changed were not the same and cannot be explained by simple scaling functions based on the thickness. This suggests that the membrane chemistry or another underlying property might play a role in the carbonation of AEMFCs.

### 3.2. Effect of AEM Chemical Structure on the Performance of AEMFCs Operating with 400 ppm CO_2_

**Figure 3:**
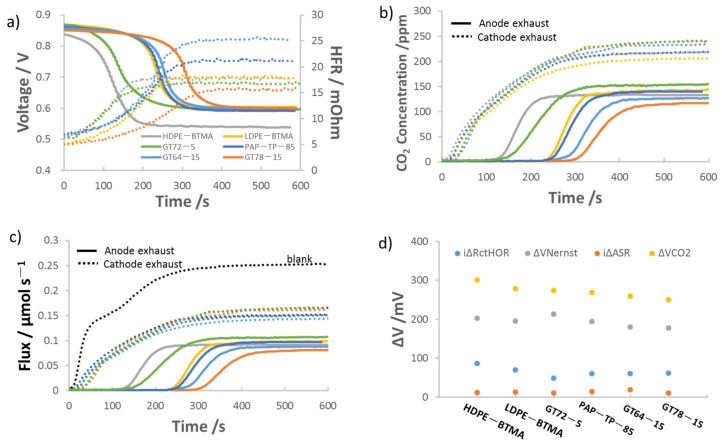
AEMFC carbonation and performance loss for six AEMs. AEMFC operating at 0.2 A/cm^2^ and 52 °C/54 °C/60 °C with 15 μm ± 2 μm HDPE-BTMA, LDPE-BTMA, GT72-5, PAP-TP-85, GT64-15 and GT78-15 AEMs, 400 ppm CO_2_ fed to cathode at t = 0 s, 1 L/min H_2_/O_2_ flowrate, 0.6 mg_Pt_ cm^−2^ ± 0.1 mg_Pt_ cm^−2^ on anode, 0.7 mg_Pt_ cm^−2^ ± 0.1 mg_Pt_ cm^−2^ on cathode. (**a**) Voltage loss (solid lines) and HFR increase (dotted lines) following the introduction of CO_2_ into the cathode; (**b**) Concentration of CO_2_ in the anode (solid lines) and cathode (dotted lines) effluent streams; (**c**) CO_2_ flux leaving the anode and cathode; (**d**) deconvolution of the CO_2_-related voltage losses.

Six AEMs—GT64-15, GT72-5, GT78-15, LDPE-BTMA, HDPE-BTMA and PAP-TP-85—were employed to investigate the effect of chemical structure on AEMFC carbonation. These anion exchange membranes were selected because they have a wide range of properties and backbones, although they are similar in thickness at 10–15 μm. The behavior of AEMFCs using these AEMs under CO_2_ dosing is presented as Figure 3. Of the membranes selected, HDPE-BTMA stood out as the fastest to reach the new steady-state voltage and HFR in Figure 3a. Compared within the same backbone and functional group, Figure 3a shows GT78-15 and LDPE-BTMA are slower to reach the new steady-state voltage and HFR than GT64-15 and HDPE-BTMA, respectively, implying that the carbonate interactions might be more complex. The former AEMs have larger HFR changes after CO_2_ dosing, suggesting GT78-15 is more resistant to CO_2_ poisoning than GT64-15 and LDPE-BTMA is slightly more resistant to CO_2_ than HDPE-BTMA.

Also, as the degree of crosslinking of the GTXX-YY series of polymers was changed, differences in the carbonation behavior were observed. For a lower degree of crosslinking and similar IEC (e.g., GT72-5 vs. GT78-15), the poly(norbornene) polymers allow for more water uptake and have higher conductivity [36,50]. This is because AEMs with less crosslinking have more degrees of freedom for movement during synthesis, allowing for their structured water and ion transport channels to be more freely established [51]. Thus, GT72-5 showed more rapid voltage and HFR stabilization after feeding CO_2_ than the other two anion exchange membranes with 15% crosslinker. This is also consistent with what was observed with PAP-TP-85. Because the PAP-TP-85 has its functional sites on the polymer backbone, they have less rotational freedom than some of the other polymers, also leading to longer equilibration times—although the same feature means that PAP-TP-85 has the best mechanical properties among the six AEMs, such as Young’s modulus and stress at break as shown in Table 1. 

**Figure 4:**
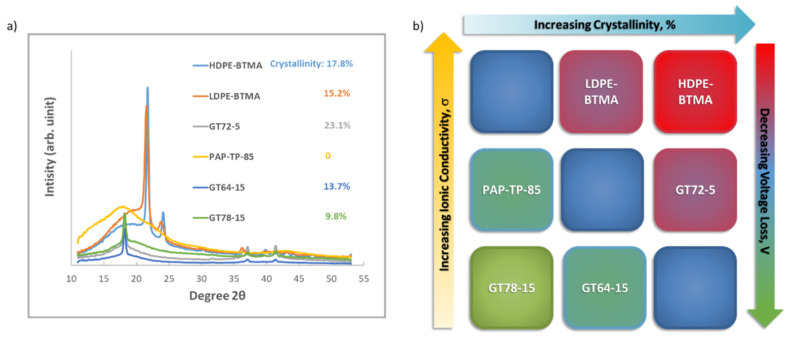
(**a**) Wide-angle X-ray scattering (WAXS) analysis of polyethylene-based, poly(norbornene) copolymer and poly (aryl piperidinium)-based AEMs; (**b**) AEMs mapping based on their relative conductivity and crystallinity properties.

The trends in the time that it takes for the cell voltage and HFR to achieve steady state after CO_2_ addition suggests that the AEM crystallinity may play a role in in its carbonation behavior. Therefore, the crystallinity of the AEMs was investigated by wide-angle X-ray scattering (WAXS) and the results are shown in in Figure 4a. The integration and quantification of membrane crystallinity is shown in the Supporting Information, Figures S13–S19. From data in Figure 4a, the degree of crystallinity obeys the following order: GT72-5 > HDPE-BTMA > LDPE-BTMA > GT64-15 > GT78-15 > PAP-TP-85. Regarding the trend, in the GT series, it makes sense that lower IEC, less cross-linked polymers would result in higher degrees of crystallinity. It also follows logically that HDPE has higher crystallinity than LDPE, though they are both similarly structured. Finally, it was observed that the PAP-TP-85 AEM is completely amorphous while still showing good conductivity and water uptake (Table 1)—showing that crystallinity and conductivity might need to be considered separately, which is discussed later. 

This leaves open the question of whether or not crystallinity is the sole descriptor for carbonation behavior. To answer this, in addition to the operating voltage and HFR, the dynamics of cell carbonation can be observed in Figure 3b, which shows CO_2_ concentration profiles of the anode and cathode exhaust for AEMFCs with all six AEMs. These are converted to the molar fluxes of CO_2_ in Figure 3c. Figure 3b shows that the (bi)carbonate transport and stabilization is indeed slower for the GT78-15 and LDPE-BTMA AEMs than the GT64-15 and HDPE-BTMA AEMs, respectively. This further suggests that the former is more CO_2_ tolerant. In addition, the CO_2_ break-through time from the anode for GT72-5 is obviously shorter than the other GT AEMs, which is consistent with the voltage decrease rate in Figure 3a. GT72-5 also has the highest anode exhaust concentration showing good CO_2_ transport ability through MEA. Next, the steady-state carbonation and CO_2_-release data in Figures S7–S12 were deconvoluted and the results are shown in Figure 3d. There, the trend in the overall CO_2_-related voltage loss was: HDPE-BTMA > LDPE-BTMA > GT72-5 > PAP-TP-85 > GT64-15 > GT78-15. Interestingly, this trend coincides better with their conductivity order—in fact, the highest conductivity AEMs showed the highest CO_2_-related voltage loss. Therefore, it appears that the best-performing cells from a carbonation perspective prefer lower crystallinity and relatively lower ionic conductivity (note that although GT78-15 and GT64-15 are classified as “lower conductivity” here, their conductivity values are all high compared to an overwhelming majority of AEMs in the literature). This is illustrated in Figure 4b. 

Digging into the individual mechanisms a bit deeper, it is not surprising that Δ*V_Nernst_* is the overwhelming contributor to the CO_2_-related voltage loss. However, both Δ*V_Nernst_* and Δ*V_ctHOR_* decreased with decreasing conductivity and increasing crystallinity, supporting the conclusion that the CO_2_ concentration gradient in these materials may extend further into the AEM.

### 3.3. Water Distribution of Carbonated AEMFC by Operando Neutron Imaging

To really see the influence of carbonation on AEMFC performance, we took the AEM that was the most influenced by carbonation (HDPE-BTMA) and exposed it to CO_2_ during *operando* neutron imaging. This was done because it has been suggested that the reaction of OH^-^ with CO_2_ can lead to a further decrease in backbone crystallinity. Therefore, the CO_2_ reaction not only affects the conductivity but also affects the overall polymer morphology. Combined, this says that the introduction of carbonate to the operating AEMFC can lower the water uptake of the AEMs [52]. However, this has never been directly measured in an operating cell. This is important because it has become well known that water content and transport is of significant importance to AEMFC performance. 

**Figure 5:**
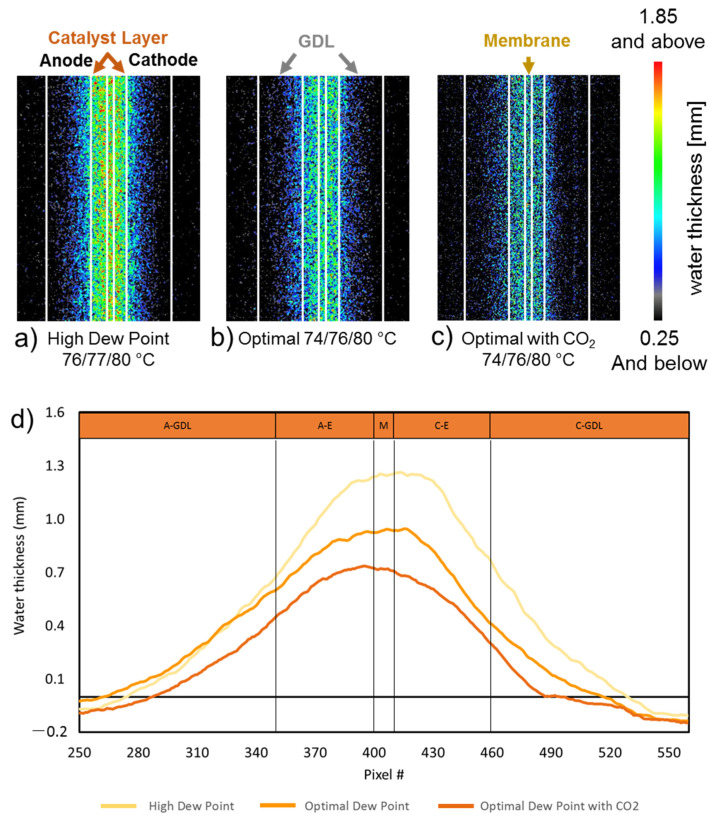
*Operando* neutron images of water in the gas diffusion layers, catalyst layers, and HDPE-BTMA AEM in operating at 1 A cm^−2^, 1 L/min at both sides, (**a**) high dew point at 76 °C /77 °C /80 °C (**b**) optimal dew point of 74 °C/76 °C/80 °C (**c**) optimal dew point and 1% CO_2_ in cathode stream (**d**) qualitative through-plane water distribution plot extracted from (**a**)–(**c**). The cells used in the neutron beam (2.5 cm^2^ active area) were constructed with a 0.88 mg_Pt_ cm^−2^ PtRu/C anode, a Pt/C cathode at 1.3 mg_Pt_ cm^−2^, and an ETFE-based AEI.

During the neutron imaging experiments, Figure 5, the cell was held at a constant current density, ensuring that the rate of water production in all experiments was identical. Therefore, any difference in the water content comes from the reacting gas dew points and water uptake abilities of the AEM + AEI. Figure 5a shows the *operando* neutron images for an AEMFC with a HDPE-BTMA AEM held at high dew points for more than 8 h without CO_2_ poisoning. Figure 5b shows the same cell at optimized dew points, also operated for 8 h without CO_2_ poisoning. Clearly, at lower dew points, the cell has less liquid water essentially everywhere in the cell due to increased convective evaporation of produced water into the reacting gases. Finally, at the optimized dew points, 1% CO_2_ was added to the cathode inlet, and the resulting neutron images are shown in Figure 5c. Compared with Figure 5b, there was significantly less water in the AEM and electrodes. Quantitative through-plane water distribution plots, Figure 5d, confirm that, indeed, introducing CO_2_ into an operating AEMFC does lower the water content of the cell, which will further decrease performance—even in addition to the three direct CO_2_-related mechanisms already recognized. 

## 4. Conclusions

In this paper, we focus on the effect of AEM properties on AEMFC carbonation which is studied for the first time. It was found that, across AEM chemistries, decreasing ionic conductivity and decreasing crystallinity tended to lower the overall CO_2_-related voltage loss. These give new design principles to manufacture next-generation AEMs with higher CO_2_ tolerance. Lastly, the content and distribution of liquid water in a stably operating AEMFC was directly observed by neutron imaging before and after carbonation. It was shown that carbonation significantly reduces the water content of the cell. 

## Figures and Tables

**Table 1:** Key properties of the polyethylene-based, poly(norbornene)-based and poly(aryl piperidinium)-based AEMs used in this study.

AEM	Cross-Linking Ratio (%)	IEC ^a^ (mmol/g)	Thickness ^b^ (μm)	WU ^c^ (%)	λ_H2O_^d^	Swelling ^e^ (%)	σ ^f^, OH^−^, 80 °C (mS cm^−1^)	Young’s Modulus (MPa) ^g^	Stress at Break (MPa) ^g^
LDPE-BTMA [35]	NR	2.54 ± 0.21	15	149 ± 16	32 ± 3	27 ± 10	208 ± 6	248 ± 31	23 ± 6
		2.87 ± 0.05	25	104 ± 9	18 ± 2	22 ± 2	145 ± 8	386 ± 83	29 ± 5
HDPE-BTMA [35]	NR	2.44 ± 0.04	15	155 ± 15	35 ± 2	38 ± 7	214 ± 2	NR	35
PAP-TP-85 [38]	NR	2.2	15	60	15.13	8	175	425	50
GT72-5 [1,37]	5	3.44	10	96	15.24	35	175	NR	NR
GT78-15 [1,37]	15	3.62	10, 20	65	9.98	50	138	NR	28
GT64-15 [1,37]	15	3.28	10	29	8.81	14	142	175	NR

^a^ IEC = mmol Cl^−^ per g (dry AEM, Cl^−^ form). ^b^ Dehydrated AEM thickness at room temperature (Cl^−^ form). ^c^ Gravimetric water uptake (Cl^−^ form) at room temperature (= 100 × (*m_hyd_*−m_dehyd_)/*m_dehyd_*, where m = mass/g). ^d^ The number of water molecules per Cl^−^ anion in the fully hydrated AEM, calculated as: **λ**_H2O_ = WU/(100 × 18.02 × IEC). ^e^ Through-plane swelling (= 100 × (*t_hyd_*−*t_dehyd_*)/*t_dehyd_*). ^f^ The 4-probe (in-plane) OH^−^ conductivity in a flowing 100% relative humidity N_2_ atmosphere. ^g^ Tensile properties of the Cl^−^-form AEMs (error within 25%) in the ambient atmosphere. NR = not reported.

**Table 2:** Degree of steady-state carbonation as function of AEM thickness of GT78-15 and LDPE-BTMA AEMs feeding with 400 ppm CO_2_.

	GT78-15		LDPE-BTMA	
	10 μm	20 μm	15 μm	25 μm
AEMFC carbonate *N_CO2_*/µmol	19 ± 1	24 ± 1	21 ± 1	32 ± 1
Δ*ASR*/mΩ·cm^2^	0.91	116	54	73
Δ*R_ctHOR_*/mΩ	46	40	65	58
Degree of Carbonation, DOC (%)	27	21	41	44

## Data Availability

Data available on request due to privacy. The data presented in this study are available on request from the corresponding author. The data are not publicly available due to privacy.

## Supplementary Materials

The following are available online at https://www.mdpi.com/2077-0375/11/2/102/s1, Figure S1: Schematic of the 2.5 cm2 active area cell used for neutron imaging. Schematic dimensions are in inch, Figure S2: Visualized calculation of mechanism deconvolution, Figure S3: Voltage and HFR changes for 10 min carbonation and 10 min recovery process. Same condition as Figure 2, Figure S4: Voltage and HFR changes for 10 min carbonation and 10 min recovery process. Same condition as Figure 2, Figure S5: Voltage and HFR changes for 10 min carbonation and 10 min recovery process. Same condition as Figure 2, Figure S6: Voltage and HFR changes for 10 min carbonation and 10 min recovery process. Same condition as Figure 2, Figure S7: Voltage and HFR changes for 10 min carbonation and 10 min recovery process. Same condition as Figure 3, Figure S8: Voltage and HFR changes for 10 min carbonation and 10 min recovery process. Same condition as Figure 3. Figure S9: Voltage and HFR changes for 10 min carbonation and 10 min recovery process. Same condition as Figure 3, Figure S10: Voltage and HFR changes for 10 min carbonation and 10 min recovery process. Same condition as Figure 3, Figure S11: Voltage and HFR changes for 10 min carbonation and 10 min recovery process. Same condition as Figure 3, Figure S12: Voltage and HFR changes for 10 min carbonation and 10 min recovery process. Same condition as Figure 3, Figure S13: Illustration of crystallinity calculation, Figure S14: Quantification of GT72-5 AEM crystallinity by convoluting WAXS and Off-axis WAXS results, Figure S15: Quantification of HDPE-BTMA AEM crystallinity by convoluting WAXS and Off-axis WAXS results, Figure S16: Quantification of LDPE-BTMA AEM crystallinity by convoluting WAXS and Off-axis WAXS results, Figure S17: Quantification of GT64-15 AEM crystallinity by convoluting WAXS and Off-axis WAXS results, Figure S18: Quantification of GT78-15 AEM crystallinity by convoluting WAXS and Off-axis WAXS results, Figure S19: Quantification of PAP-TP-85 AEM crystallinity by convoluting WAXS and Off-axis WAXS results.
