# Supplementary material for: Effect of Membrane Properties on the Carbonation of Anion Exchange Membrane Fuel Cells
Source: Membranes (Basel). 2021 Jan 31;11(2):102. doi: 10.3390/membranes11020102 (PMC7912077; doi:10.3390/membranes11020102)
Supplement: Supplementary file 1 [file membranes-11-00102-s001.pdf]

membranes

Article

Effect of Membrane Properties on the Carbonation of Anion Exchange Membrane Fuel Cells

Yiwei Zheng [1], Lyzmarie Nicole Irizarry Colón [1], Noor Ul Hassan [1], Eric R. Williams [2], Morgan Stefik [2], Jacob M. LaManna [3], Daniel S. Hussey [3] and William E. Mustain [1,*]

[1] Department of Chemical Engineering, Swearingen Engineering Center, University of South Carolina, Columbia, SC 29208, USA; yiweiz@email.sc.edu (Y.Z.); lyzmarie@email.sc.edu (L.N.I.C.); ulhassan@email.sc.edu (N.U.H.)
[2] Department of Chemistry and Biochemistry, University of South Carolina, Columbia, SC 29208, USA; ericrw@email.sc.edu (E.R.W.); stefik@mailbox.sc.edu (M.S.)
[3] National Institute for Standards and Technology, Gaithersburg, MD 20899, USA; jacob.lamanna@nist.gov (J.M.L.); daniel.hussey@nist.gov (D.S.H.)
* Correspondence: mustainw@mailbox.sc.edu; Tel.: +1-803-576-6393

Citation: Zheng, Y.; Colón, L.N.I.; Hassan, N.U.; Williams, E.R.; Stefik, M.; LaManna, J.M.; Hussey, D.S.; Mustain, W.E. Effect of Membrane Properties on the Carbonation of Anion Exchange Membrane Fuel Cells. *Membranes* **2021**, *11*, 102. https://doi.org/10.3390/membranes11020102

Academic Editor: Hsiharng Yang
Received: 13 January 2021
Accepted: 26 January 2021
Published: 31 January 2021

Publisher's Note: MDPI stays neutral with regard to jurisdictional claims in published maps and institutional affiliations.

Copyright: © 2021 by the authors. Submitted for possible open access publication under the terms and conditions of the Creative Commons Attribution (CC BY) license (http://creativecommons.org/licenses/by/4.0/).

Flowfield Demensions for the *Operando* Neutron Imaging Experiments at NIST

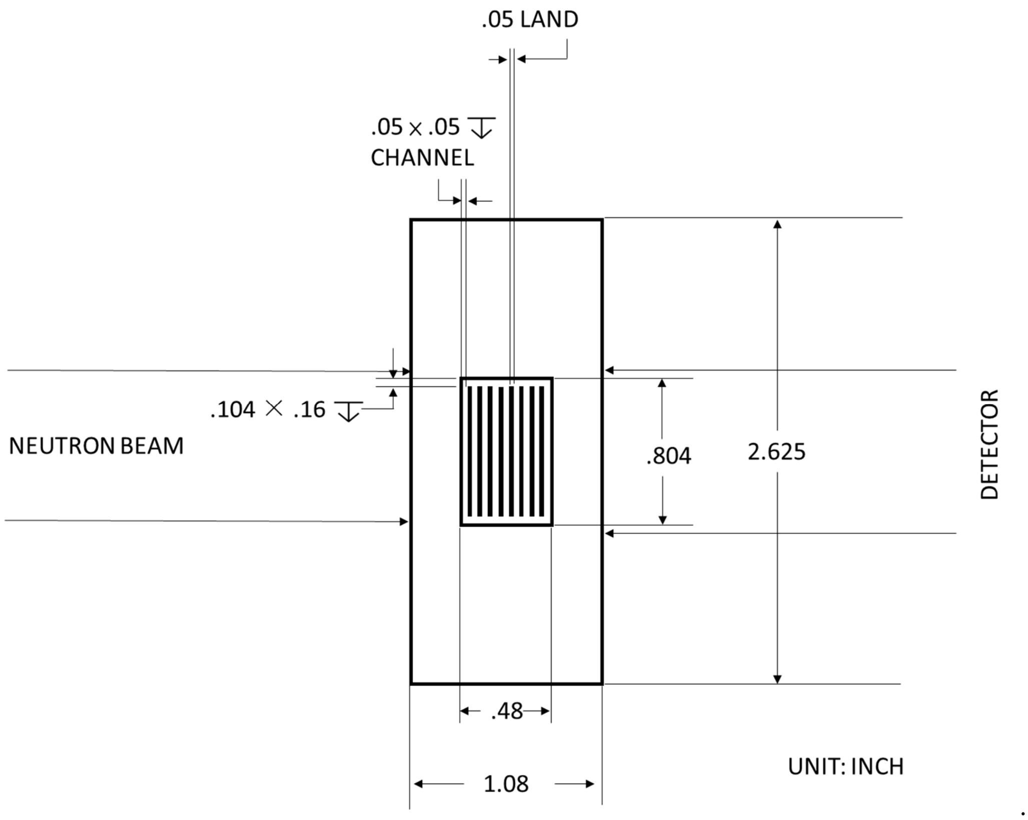

Figure S1. Schematic of the 2.5 cm^2 active area cell used for neutron imaging. Schematic dimensions are in inch.

Discussion and Calculations for AEMFC Carbonation

The total amount of carbonate (N_{CO2}) in Table 2 was calculated by first converting the CO_2 concentration in the anode and cathode effluents to fluxes (Figure 2c). Then, the area under each curve was found by integration. The same was done for a "blank" condition, was achieved by placing a Teflon membrane which does not uptake carbonate at all between the flowfields with no electrodes [1,2]. The "blank" curve accounts for the bubbler equilibration time also. Finally, the areas from the anode and cathode exhaust are subtracted from the area under the "blank" curve to yield N_{CO2}, Equation (S1).

$$N_{CO2} = \int \text{"blank"(t) dt} - \int \text{"anode exhaust"(t) dt} - \int \text{"cathode exhaust"(t) dt} \quad (1)$$

The "blank" experiment was done by placing a Teflon membrane between the flowfields with no electrodes. Its zero carbonate uptake assists a real CO_2 concentration measurement of the CO_2 gas bubbler at various conditions. It implies the total amount of CO_2 dosing into the system. Hence, the quantity of CO_2 that has been taken up into the cell by AEM and AEI can be calculated by Equation (S1). The blank curve accounts for awakening time and the time lag due to CO_2 saturated with water, leading to a bump shape of its curve within the first 100s.

Concentration was converted to dynamic flux change as presented in Figure 2c. Maintaining the mass balance, corrected by flowrate difference and reaction consumption, flux evolution shows the same conclusions as Figure 2b. The conversion is shown as equation below:

$$\text{Flux}\left[\frac{\text{umol}}{\text{s}}\right]=\frac{\text{Concentration [ppm]} * \rho[\text{g/ml}]}{Mw\left[\frac{g}{mol}\right] * 60[s/min]} * Flowrate\left[\frac{L}{min}\right]$$

$$* \ reaction\ comsuption\ corrector$$

Reaction consumption corrector is given an example at 1A/cm² (5A), for HOR at anode:

$$\text{reaction consumption corrector} = 1 - \frac{5\left[\frac{\text{C}}{\text{s}}\right] * 60\left[\frac{\text{s}}{\text{min}}\right] * 22.4\left[\frac{L}{mol}\right]}{2 * 96458\left[\frac{\text{C}}{\text{mol}}\right] * 1\left[\frac{L}{min}\right]} = 0.965$$

The procedure of decoupling three mechanisms are shown as below. In Figure S1, the voltage increase during 10 min establishment of the new quasi steady-state after CO_2 removed was mostly attributed to the relaxation of the kinetic limitations described by ΔV_{ctHOR} (ΔR_{ctHOR}). 1.0 A cm^{-2} with 400 ppm CO_2 in the cathode, anode and cathode flowrate both at 1 L/min, ΔR_{ctHOR} was calculated by equations below:

$$\Delta V_{ctHOR}(\text{mV}) = [0.513\text{ V} - 0.406\text{ V}] \times 1000 - (1.0\text{ A cm}^{-2})(5\text{ cm}^2)(23.94\text{ m}\Omega\text{ cm}^2 - 21.64\text{ m}\Omega\text{ cm}^2) = 95.5\text{ mV}$$

$$\Delta R_{ctHOR}(\text{m}\Omega) = \frac{95.5\text{ mV}}{(1.0\text{ A cm}^{-2})(5\text{ cm}^2)} = 19.1\text{ m}\Omega$$

As Equation 4 in main text, Nernstian voltage loss is described below:

$$\Delta V_{\text{Nernst}} = \Delta V_{\text{CO2}} - \Delta V_{ctHOR} - \Delta V_{\text{ASR}} = [0.67\text{ V} - 0.406\text{ V}] \times 1000 - 95.5\text{ mV} - (1.0\text{ A cm}^{-2})(5\text{ cm}^2)(23.94\text{ m}\Omega\text{ cm}^2 - 17.58\text{ m}\Omega\text{ cm}^2) = 136.7\text{ mV}$$

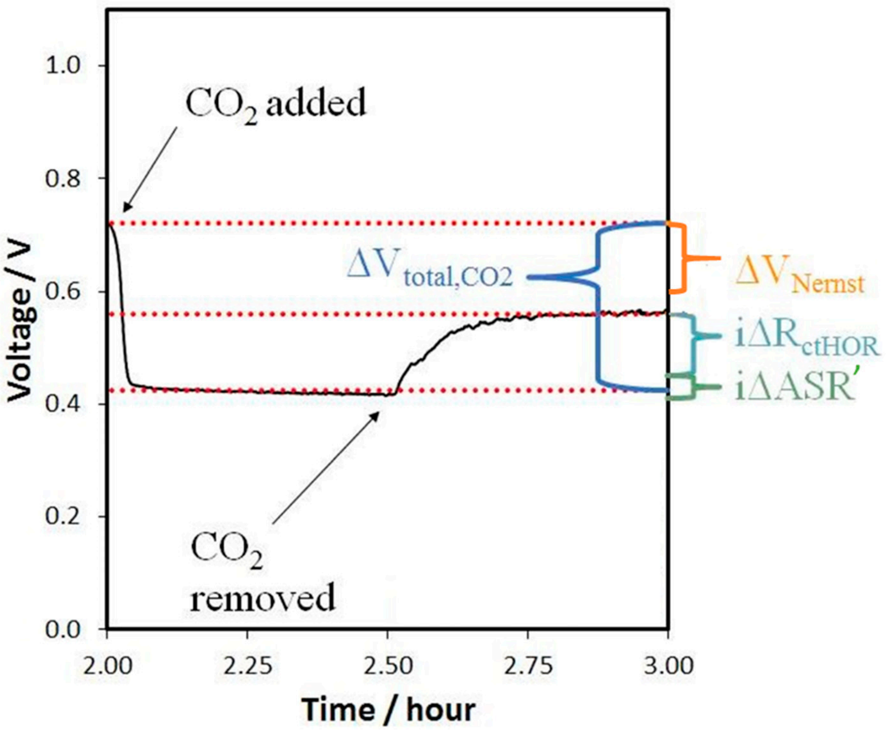

Figure S2. Visualized calculation of mechanism deconvolution.

The degree of carbonation (DOC, % of charge groups converted to the carbonate form) can be calculated by:

Total concentration of charge groups for HDPE-BTMA as an example in Figure 3:

$$DOC = \frac{(Charge\ per\ CO_2)(\mu mol\ CO_2)}{(Charge\ per\ group)(\mu mol\ AEM\ groups + \mu mol\ AEI\ groups)}$$

$$= \frac{(2)(15)}{(1)(46.0 + 52.2)} = 0.31\%$$

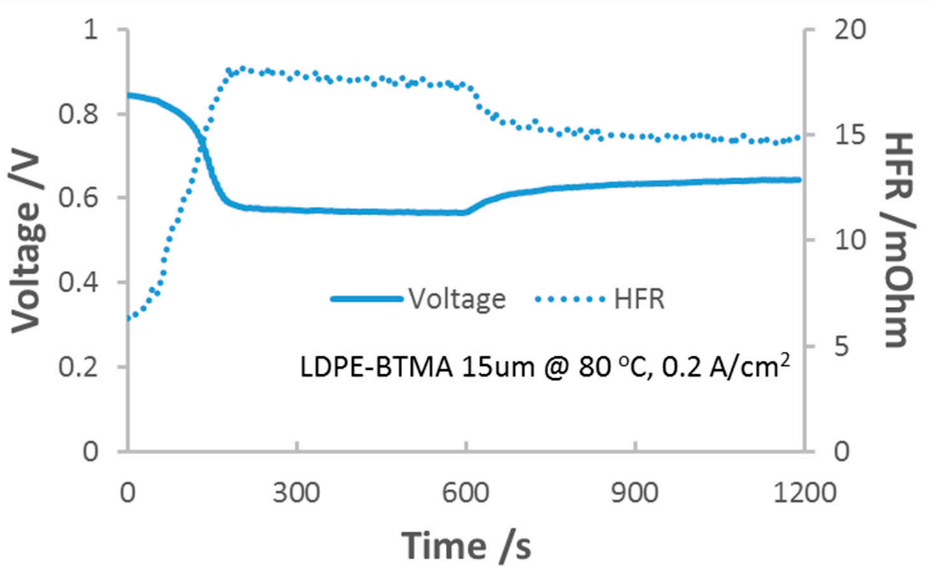

Figure S3. Voltage and HFR changes for 10 min carbonation and 10 min recovery process. Same condition as Figure 2.

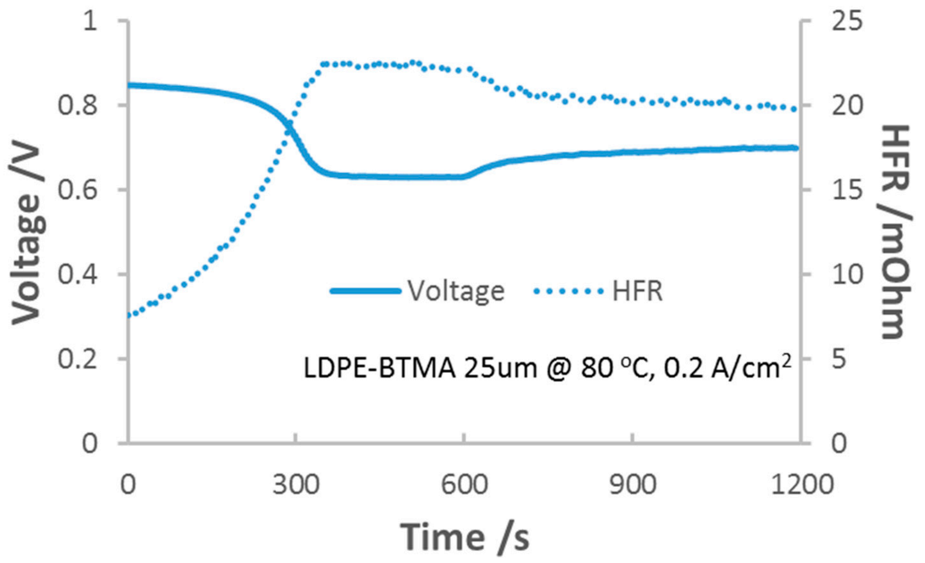

Figure S4. Voltage and HFR changes for 10 min carbonation and 10 min recovery process. Same condition as Figure 2.

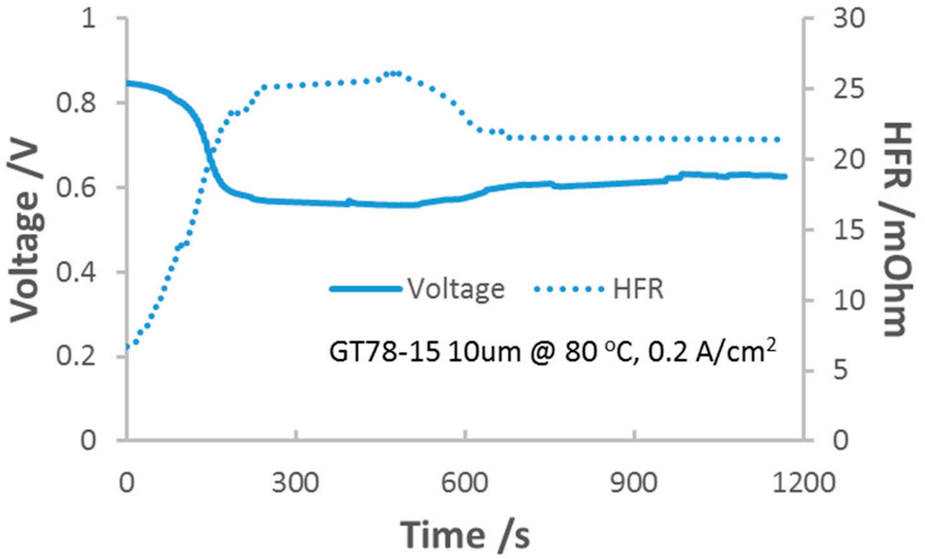

Figure S5. Voltage and HFR changes for 10 min carbonation and 10 min recovery process. Same condition as Figure 2.

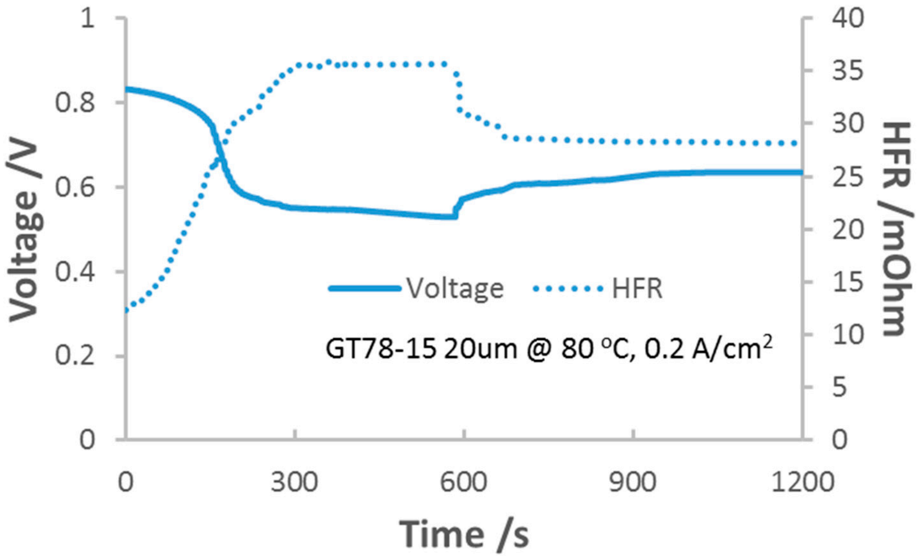

Figure S6. Voltage and HFR changes for 10 min carbonation and 10 min recovery process. Same condition as Figure 2.

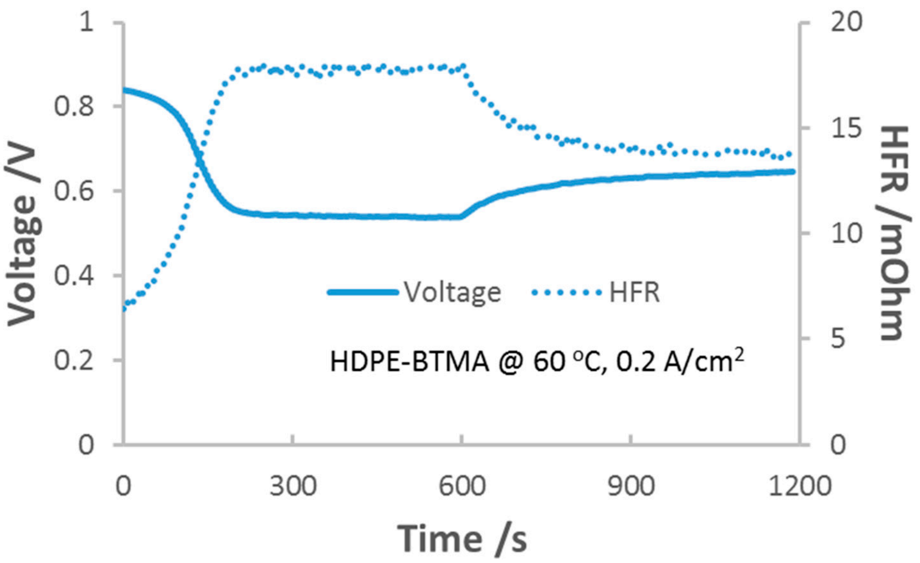

Figure S7. Voltage and HFR changes for 10 min carbonation and 10 min recovery process. Same condition as Figure 3.

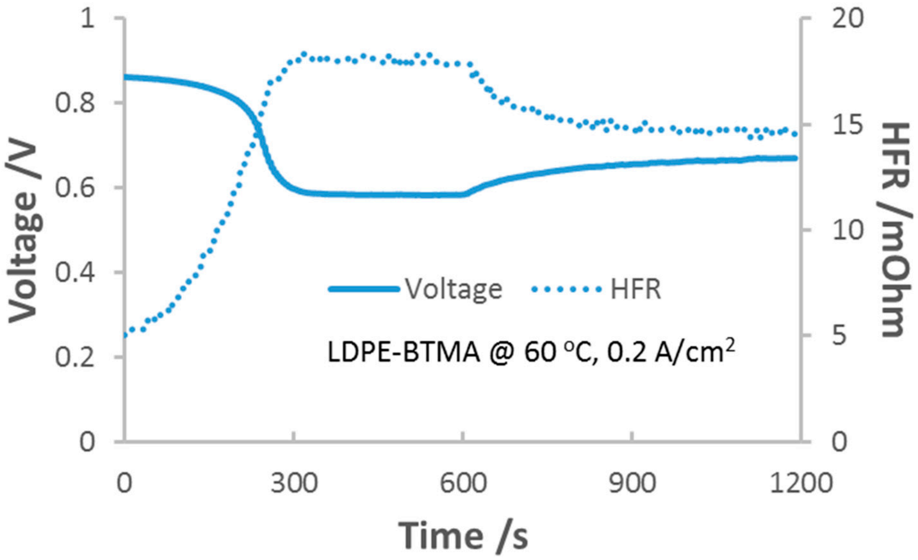

Figure S8. Voltage and HFR changes for 10 min carbonation and 10 min recovery process. Same condition as Figure 3.

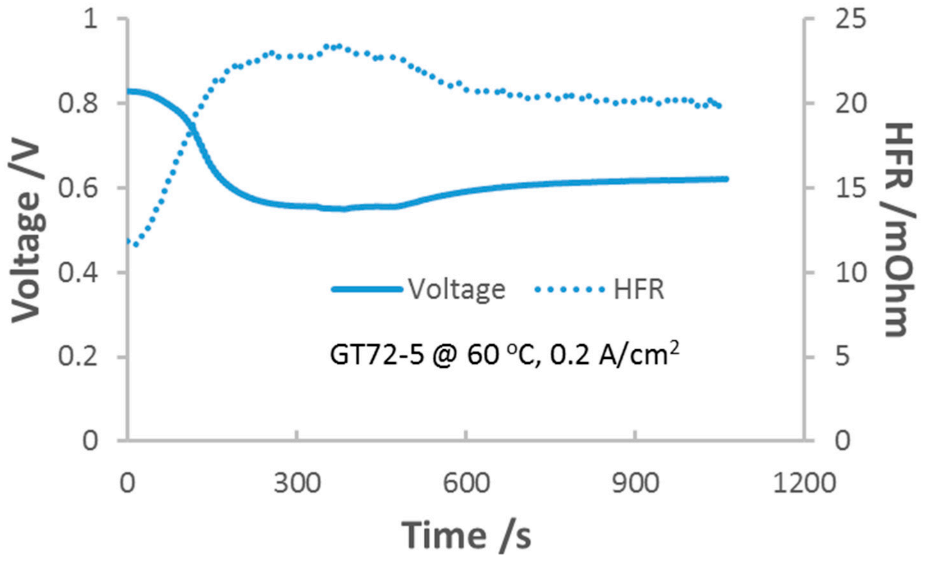

Figure S9. Voltage and HFR changes for 10 min carbonation and 10 min recovery process. Same condition as Figure 3.

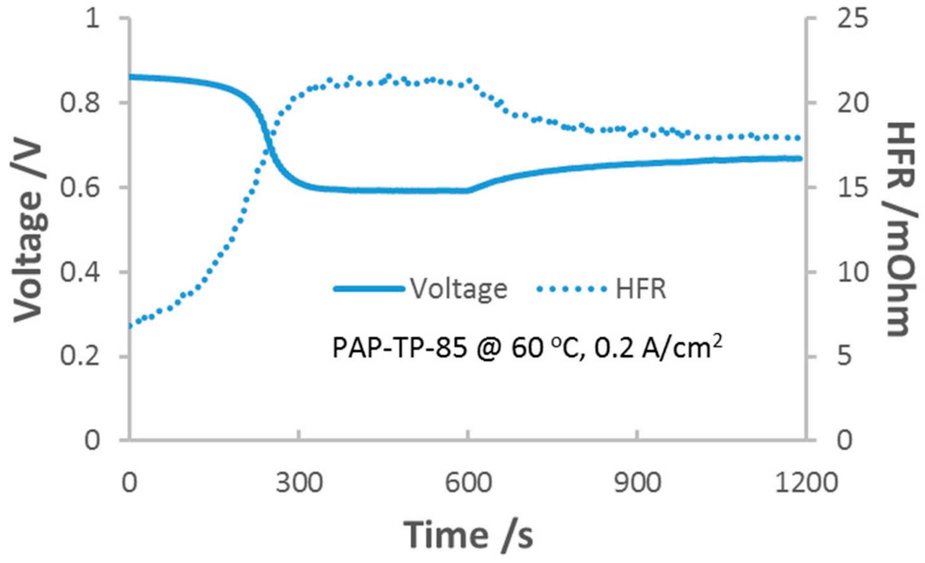

Figure S10. Voltage and HFR changes for 10 min carbonation and 10 min recovery process. Same condition as Figure 3.

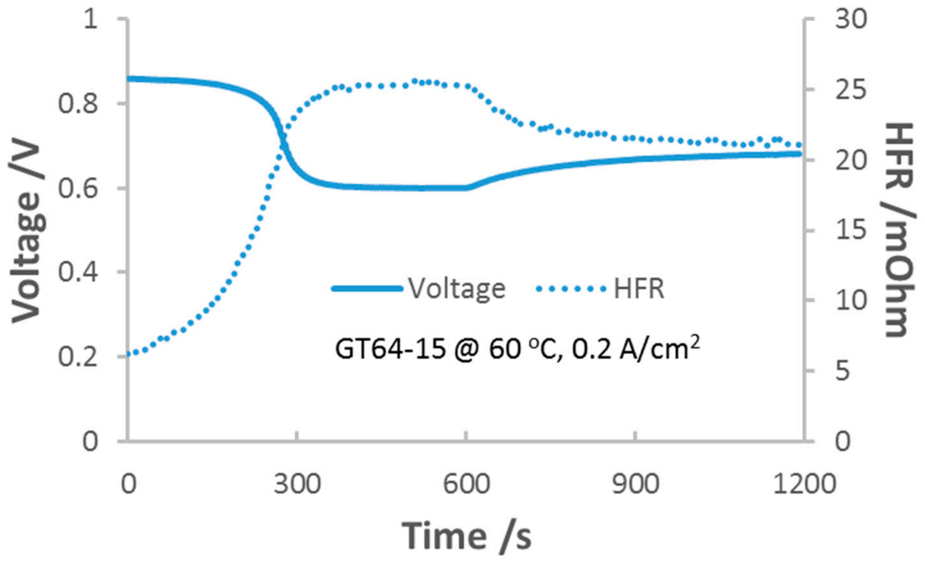

Figure S11. Voltage and HFR changes for 10 min carbonation and 10 min recovery process. Same condition as Figure 3.

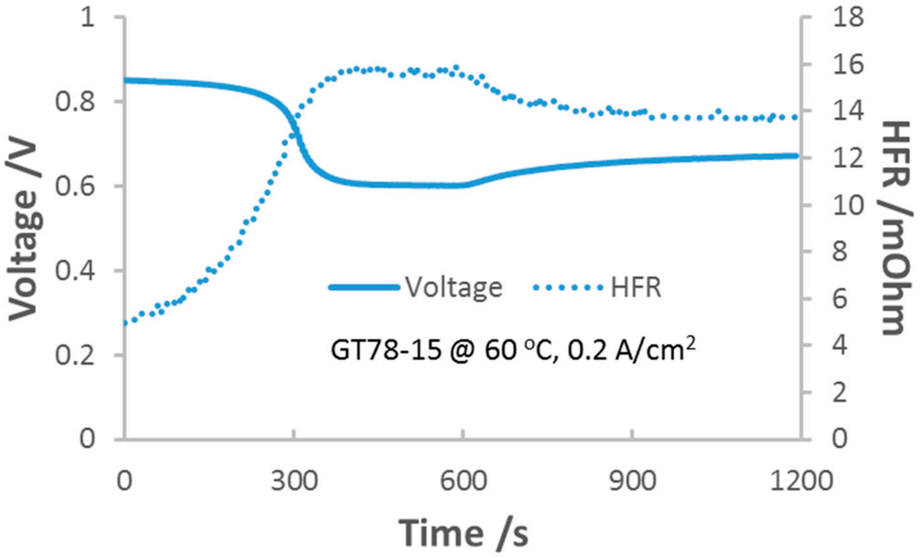

Figure S12. Voltage and HFR changes for 10 min carbonation and 10 min recovery process. Same condition as Figure 3.

Details for Wide Angle X-Ray Scattering and Calculations

The measurement of polymer crystallinity is defined as:

$$\%\ Crystallinity = \frac{X_c}{X_c + X_a} \times\ 100$$

where X_c and X_a refer to the area of the crystalline and amorphous regions of scattering, respectively.

Shown as Figure S13, crystalline and amorphous regions were deconvolved by fitting crystalline regions to a linear baseline. The crystalline integral was defined as the area above this baseline while the amorphous was the region below. In other words, the amorphous integral is the total convolved integral minus the crystalline integral fit to a linear baseline. Integral values provided are arbitrary, but their relationship provides the basis for determination of percent crystallinity for the sample. Figure S3-8 show WAXS results for GT72-5, HDPE-BTMA, LDPE-BTMA, GT64-15, GT78-15, PAP-TP-85 AEMs.

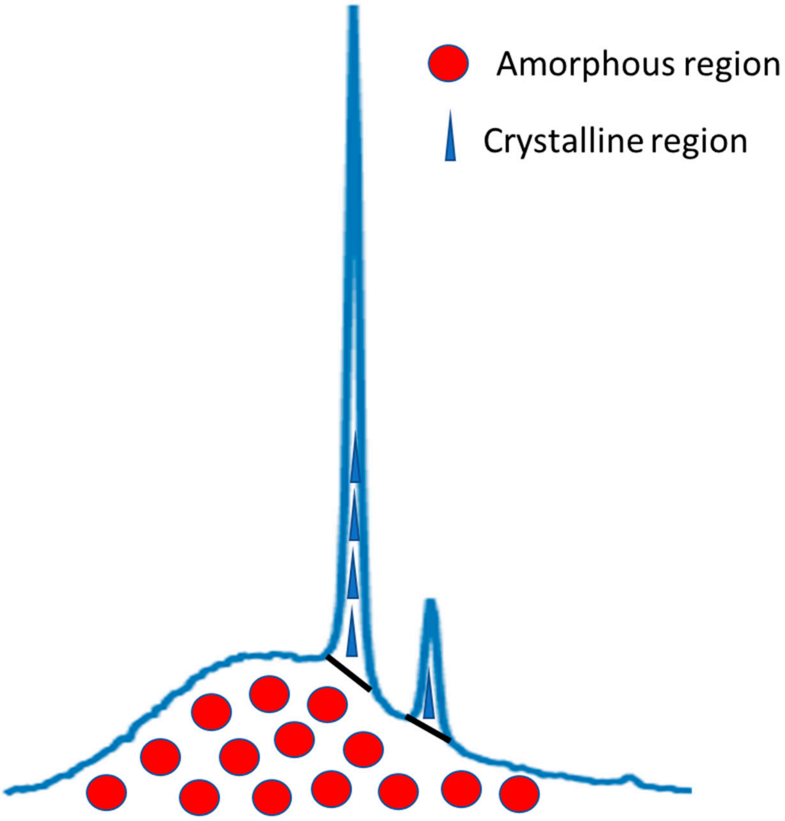

Figure S13. Illustration of crystallinity calculation.

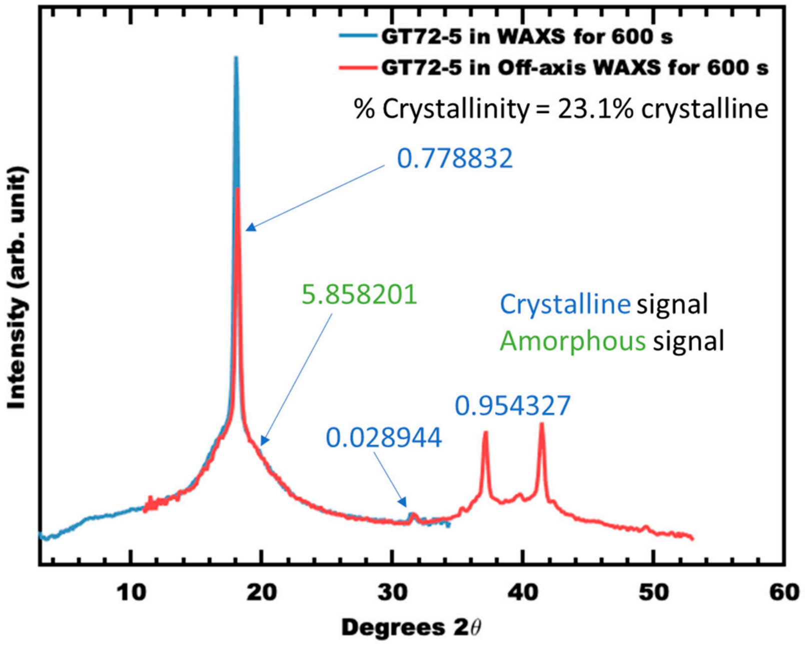

Figure S14. Quantification of GT72-5 AEM crystallinity by convoluting WAXS and Off-axis WAXS results.

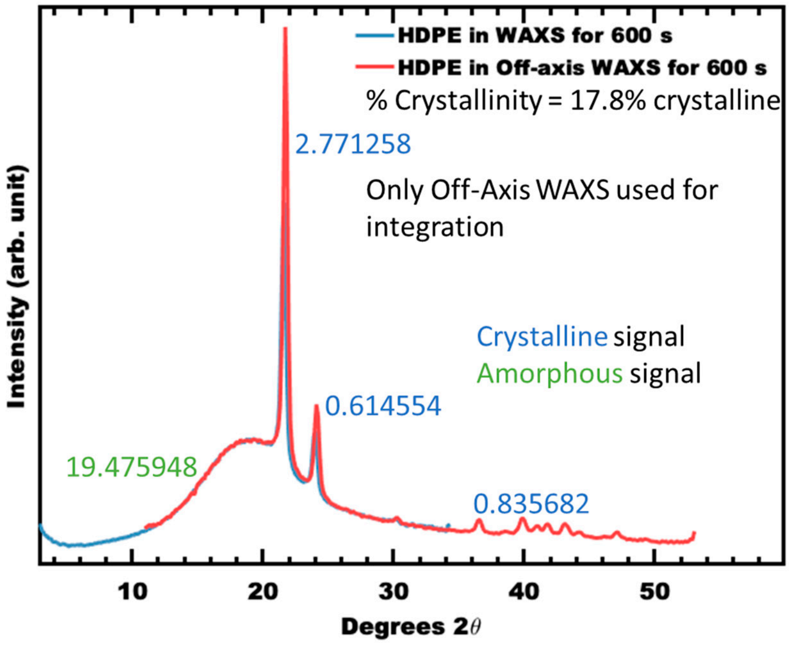

Figure S15. Quantification of HDPE-BTMA AEM crystallinity by convoluting WAXS and Off-axis WAXS results.

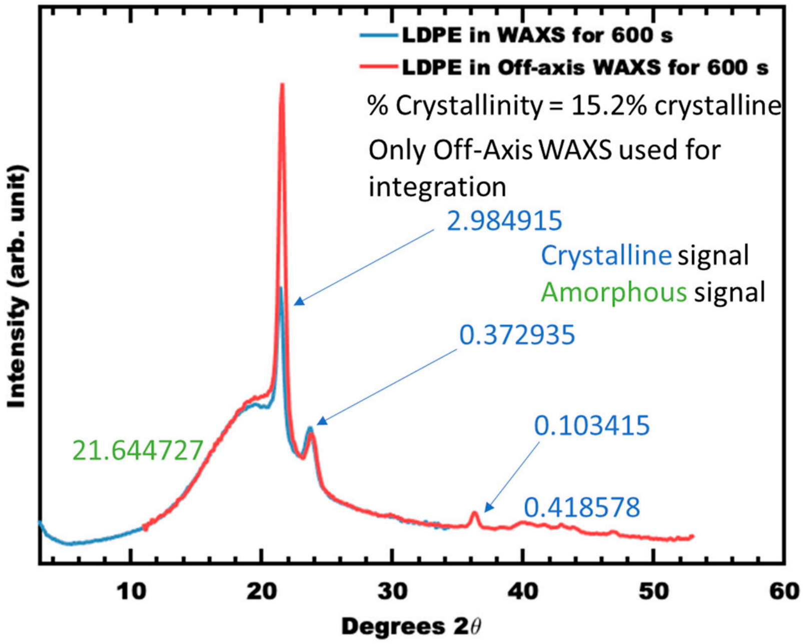

Figure S16. Quantification of LDPE-BTMA AEM crystallinity by convoluting WAXS and Off-axis WAXS results.

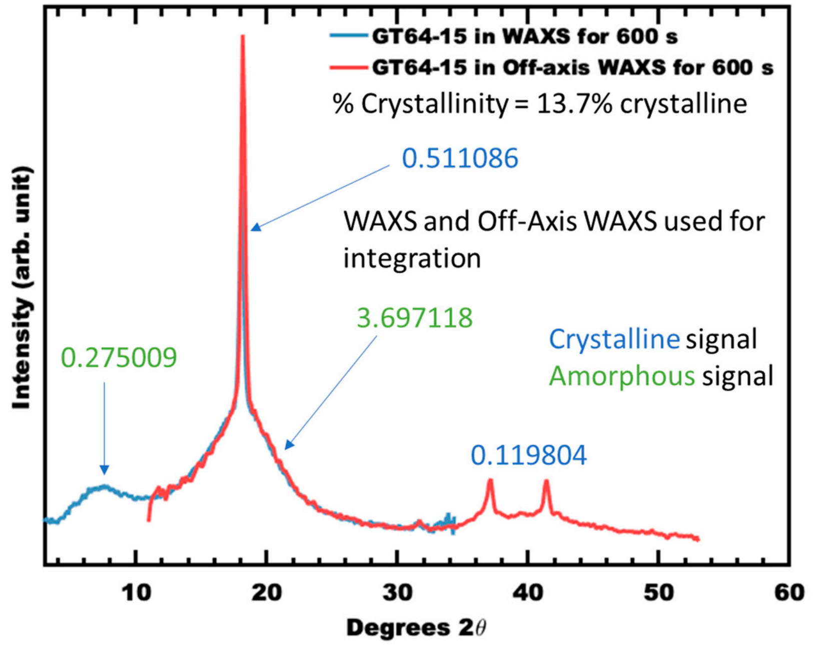

Figure S17. Quantification of GT64-15 AEM crystallinity by convoluting WAXS and Off-axis WAXS results.

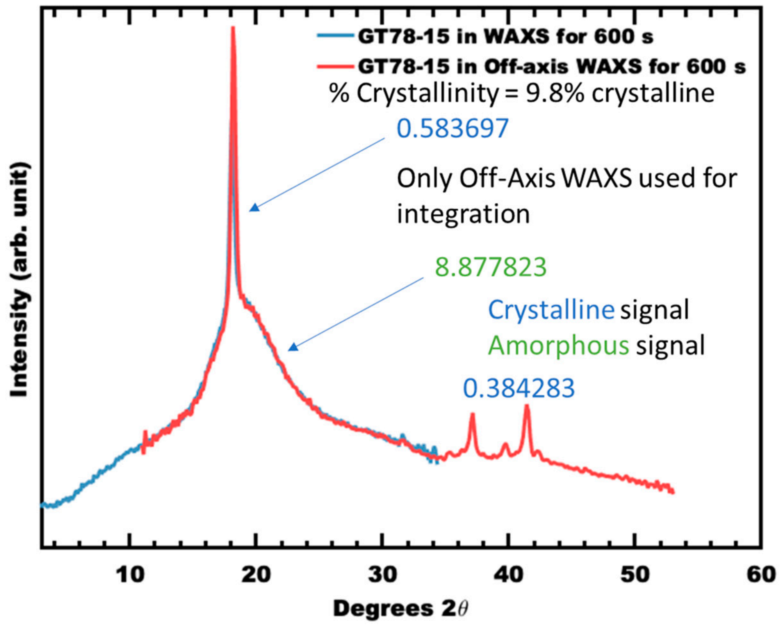

Figure S18. Quantification of GT78-15 AEM crystallinity by convoluting WAXS and Off-axis WAXS results.

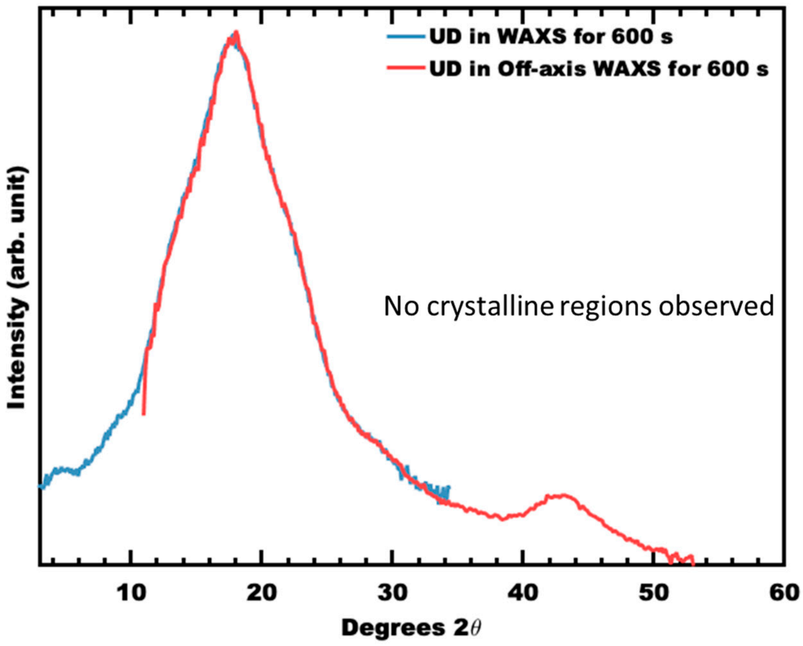

Figure S19. Quantification of PAP-TP-85 AEM crystallinity by convoluting WAXS and Off-axis WAXS results.

References

1. Y. Zheng, T. J. Omasta, X. Peng, L. Wang, J. R. Varcoe, S. Pivovar, and W. E. Mustain, *Energy Environ. Sci.*, **2019**, **12**, 2806–2819
2. Y. Zheng, G. Huang, L. Wang, J. R. Varcoe, P. A. Kohl, and E. Mustain, *J. Power Sources*, **2020**, **467**, 228350.
